# Risk and prognosis of second corpus uteri cancer after radiation therapy for pelvic cancer: A population-based analysis

**DOI:** 10.3389/fonc.2022.957608

**Published:** 2022-09-29

**Authors:** Guanhua Yu, Ran Wei, Shuofeng Li, Yongjiao Wang, Hengchang Liu, Tianli Chen, Xu Guan, Xishan Wang, Zheng Jiang

**Affiliations:** ^1^ Department of Colorectal Surgery, National Cancer Center/National Clinical Research Center for Cancer/Cancer Hospital, Chinese Academy of Medical Sciences and Peking Union Medical College, Beijing, China; ^2^ Community Health Service Center, Zaoyuan Sub-District Office, Jinan, China

**Keywords:** pelvic cancers, second corpus uteri cancer, radiotherapy, prognostic factor, overall survival

## Abstract

**Background:**

Radiation therapy (RT) is a standard treatment for the local control of primary pelvic cancers (PPC), yet the risk of second corpus uteri cancer (SCUC) in PPC patients undergoing RT is still controversial. This study investigated the impact of RT on the risk of SCUC and assessed the survival outcome.

**Methods:**

We queried nine cancer registries for PPC cases in the Surveillance, Epidemiology, and End Results (SEER) database. The cumulative incidence of SCUC was analyzed using Cox regression and Fine–Gray competing risk regression analysis. The Poisson regression analysis was employed to assess the standardized incidence ratios (SIRs) and radiation-attributed risk (RR) for SCUC. We evaluated the overall survival of patients with SCUC using the Kaplan–Meier method.

**Results:**

Receiving radiotherapy was strongly associated with a higher risk of developing SCUC for PPC patients in Fine–Gray competing risk regression (No-RT vs. RT: adjusted HR = 1.77; 95% CI, 1.40–2.28; *p* < 0.001). The incidence of SCUC in PPC patients who received RT was higher than in the US general population (SIR, 1.66; 95% CI, 1.41–1.93; *p* < 0.05), but the incidence of SCUC in patients who did not receive RT was lower than with the US general population (SIR, 0.68; 95% CI, 0.61–0.75; *p* < 0.05). The dynamic SIR and RR for SCUC decreased with decreasing age at PPC diagnosis and decreased with time progress. In terms of overall survival, 10-year survival rates with SCUC after No-RT (NRT) and SCUC after RT were 45.9% and 25.9% (HR = 1.82; 95% CI, 1.46–2.29; *p* < 0.001), respectively.

**Conclusion:**

Radiotherapy for primary pelvic cancers is associated with a higher risk of developing SCUC than patients unexposed to radiotherapy. We suggest that patients with pelvic RT, especially young patients, should receive long-term monitoring for the risk of developing SCUC.

## Introduction

Currently, radiation therapy (RT) is one of the main components of multimodality therapy for cancer management. RT has been extensively utilized alone or combined with surgery and chemotherapy to treat a variety of cancers (1). More than half of the patients with malignant tumors undergo RT with curative or palliative intents during their course of illness (2). RT plays a crucial role in improving disease control by precisely depositing high physical energy radiations on cancer cells, subsequently resulting in DNA damage and apoptosis of cancer cells (3).

However, RT is a double-edged sword. As with any cancer therapy, RT has short-term and long-term side effects which limit treatment applicability and affect patient survival (4–8). A second primary cancer occurrence is regarded as a severe event among long-term cancer survivors who underwent RT. Rombouts reported an increased risk of second rectal cancer after RT for pelvic cancers (9). Similarly, Warschkow demonstrated the linkage between pelvic RT for primary rectal cancer and the occurrence of second cancers including endometrial cancer (10). OHNO also found that patients who were diagnosed with cervical cancer and underwent RT had a small but significantly higher incidence of second cancer (11). In addition, Liauw et al. revealed that RT for primary prostate cancer was correlated with an increased risk of developing second malignancies (12). Moreover, there are multiple other studies that suggested that patients receiving RT for primary pelvic cancers (PPC) are at an increased risk of second malignancies in adjacent organs like the rectum, bladder, and prostate (13–15). It is worth noting that the uterus is in a close anatomical relationship with other pelvic target organs, which is within the pelvic irradiation field. Consequently, the uterus receives relatively high doses of irradiation during RT for one of the pelvic organs. Although several studies have assessed the role of pelvic RT in the development of second malignancies in rectum, prostate and other pelvic organs (16–20), data on the association between the risk of secondary corpus uteri cancer (SCUC) and RT for PPC are still lacking (21). Whether or not RT for PPC is associated with increased risk of SCUC remains debatable. Hence, we used data obtained from the National Cancer Institute’s Surveillance, Epidemiology, and End Results (SEER) database to comprehensively investigate the influence of RT for PPC on the risk of SCUC and to evaluate the long-term prognosis.

## Methods

### Database and study population

We identified female patients diagnosed with solid pelvic cancers in five sites as their initial primary malignancies from the nine registries (Atlanta, Connecticut, Detroit, Hawaii, Iowa, New Mexico, San Francisco–Oakland, Seattle–Puget Sound, and Utah) of the SEER program between January 1975 and December 2018. The pelvic cancers consisted of the rectum and rectosigmoid, anorectum, cervix uteri, ovary, and bladder, which were included based on the third edition of the International Classification of Diseases for Oncology (ICD-O-3). The PPCs in regional and localized stages identified by SEER were collected for analysis. The exclusion criteria included patients who were not diagnosed with the first primary pelvic cancer, were younger than 20 years old, had a survival and follow-up time of less than 12 months after PPC diagnosis, had no surgery, distant metastases, were treated with radioisotopes and radioactive implants, and had missing data on race surgery performed, radiation, chemotherapy, tumor stage and grade, age and follow-up information. This study has been approved by the Ethics Committee of Cancer Hospital, Chinese Academy of Medical Sciences.

### Treatment interventions

Patients with pelvic cancers, including tumors in the rectum and rectosigmoid, anorectum, cervix uteri, ovary, and bladder, are routinely treated with beam radiation. Patients would be divided into two groups based on their radiation therapeutic schedule: No-radiotherapy (NRT) and RT. The dosages of radiation administered and detailed adjuvant radiotherapy treatment was not registered in the SEER database.

### Outcomes

The primary endpoint was to evaluate the risk of SCUC during more than 12 months of latency time after receiving RT. The secondary primary cancers were identified with ICD-O-3 guidelines by the SEER database, which could prevent the inclusion of locoregional or distant recurrences of PPC. The latency time for the development of SCUC started at 1 year after the diagnosis time of primary PPC and ended at the date of diagnosis of SCUC, overall survival (OS), cancer-specific survival (CSS), or reaching 30 years of follow-up, whichever happened first.

The secondary outcome was to calculate the 10-year OS and CSS of SCUC. The OS time was established from the diagnosis of SCUC to the data of all-cause death, and the CSS time was established from the diagnosis of SCUC to the data of cancer-specific death. The SCUC patients were divided into two groups: SCUC patients receiving RT for primary PPC and SCUC patients without RT for primary PPC. Moreover, the patients with sporadic corpus uteri cancer and who did not develop any second cancers during their follow-up time, who were referred to as the only primary corpus uteri cancer (OPCUC) patients, were included in the survival analysis to investigate the prognostic effects of RT on SCUC.

### Statistical methods

The Cox regression analysis was performed to evaluate the risk of SCUC in primary PPC patients. However, the Fine–Gray competing risk regression analysis was also established with SCUC as the primary endpoint and other kinds of secondary primary cancer or all-cause death were considered competing events, which was performed to eliminate the influence from competing events. Both of them were performed to calculate the hazard ratios (HRs) and 95% confidence intervals (CIs) of SCUC occurrence after RT. The characteristic features of primary PPC with a *p*-value of less than 0.05 (two-sided) in the univariable analysis would be included in the multivariable risk model through the backward method.

Furthermore, we evaluated the radiotherapy-associated risk (RR), standardized incidence ratio (SIR), and 95% CIs through Poisson regression analysis. The definition of RR in our study was the ratio of SCUC occurrence in PPC patients, and the SIR was the ratio of SCUC occurrence among PPC patients to the incidence of corpus uteri cancer in the US general population. Both RR and SIR were adjusted for age at PPC diagnosis and calendar year of PPC diagnosis. The RR was evaluated with R software (version 3.5.3), and the SIR was calculated with SEER*Stat 8.3.6.

The Kaplan–Meier method and the log-rank test were performed to evaluate the 10-year OS and CSS of SCUC and OPCUC patients. To balance the characteristic baseline between SCUC and OPCUC patients, we used the propensity score matching (PSM) to match the cases with age at corpus uteri cancer (CUC) diagnosis, year of CUC diagnosis, race, stage of CUC, and type of treatment for CUC.

## Results

### Patient characteristics

**Figure 1:**
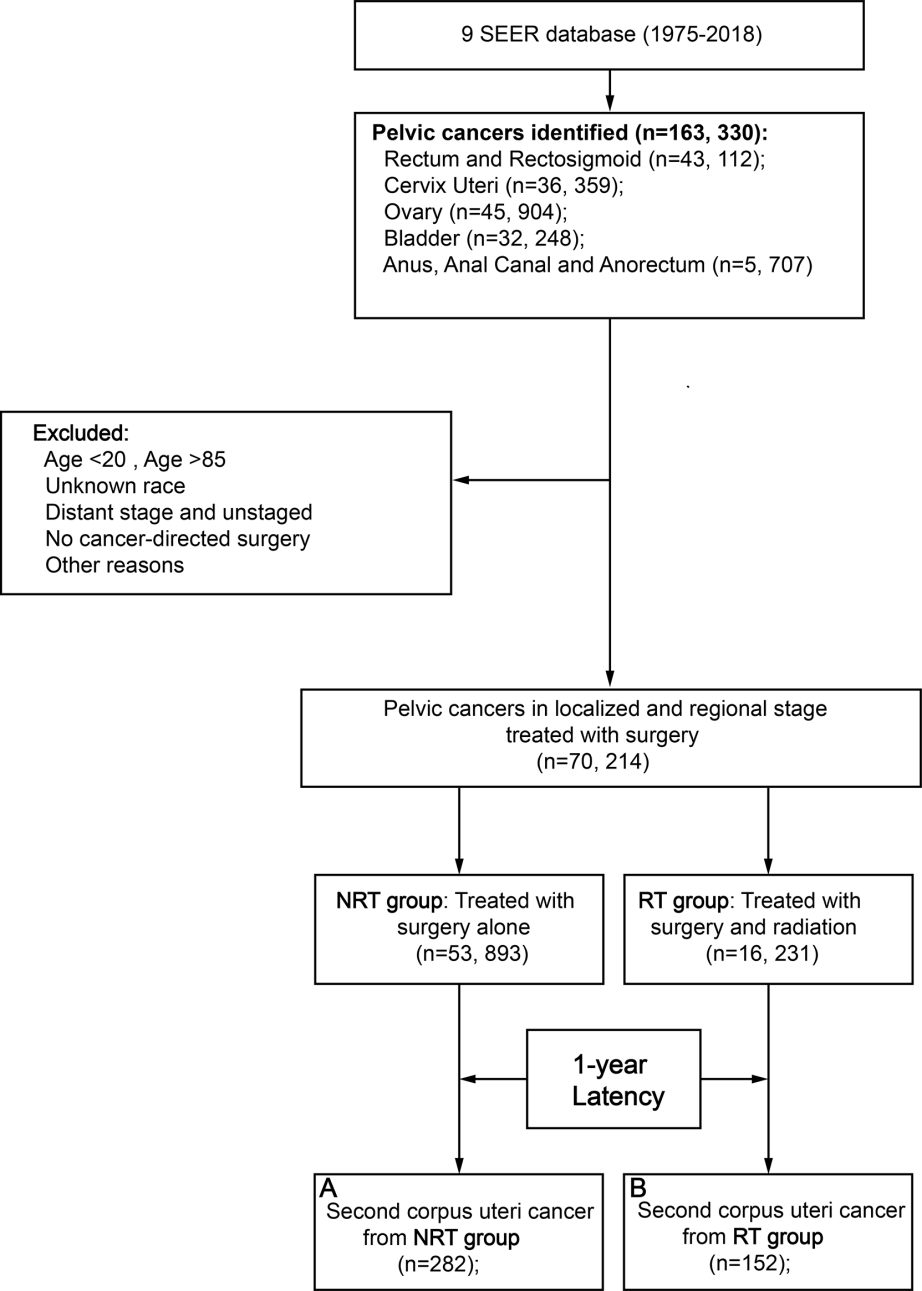
Flow diagram. RT, radiation therapy; NRT, no radiation therapy; SEER, Surveillance, Epidemiology and End Results; PSM, propensity score matching.

A total of 70,214 patients with primary pelvic cancer were included in this population-based study, 16,231 patients underwent pelvic RT and 53,983 patients did not receive pelvic RT, respectively (**Figure 1**; **Table 1**). Primary pelvic cancers include malignancies in the rectum and rectosigmoid (39%), bladder (27%), cervix uteri (17%), ovary (15%), anus, anal canal, and anorectum (2%). After 1 year of latency after PPC diagnosis, 152 patients in the RT group and 282 patients in the NRT group developed SCUC, indicating that patients with PPC in the RT group are more susceptible to SCUC. Compared with patients without RT, patients in the RT group present with younger age at PPC diagnosis, more advanced tumor grade (III/IV), and regional tumor stage, with *p* < 0.001. Consequently, a significantly higher proportion of patients in the RT group received chemotherapy in comparison to patients without RT, with *p* < 0.001. The detailed baseline characteristic of patients is shown in **Table 1**.

**Table 1:** Comparisons of Baseline Characteristics of Patients with PPC by Treatment Modality.

Characteristic	Pelvic Cancer Patients				Pelvic Cancer Patients with SCUC		
	Total	NRT	RT	P-value	NRT	RT	P-value
	N=70214	N=53983	N=16231		N=282	N=152	
**Age at PPC Diagnosis**				<0.001			0.020
20-49	16737 (0.24)	12134 (0.23)	4603 (0.28)		52 (0.18)	15 (0.10)	
50-69	29284 (0.42)	21811 (0.40)	7473 (0.46)		158 (0.56)	104 (0.68)	
≥ 70	24193 (0.34)	20038 (0.37)	4155 (0.26)		72 (0.26)	33 (0.22)	
**Year of PPC Diagnosis**				<0.001			<0.001
1975-1984	13849 (0.20)	11609 (0.21)	2240 (0.14)		87 (0.31)	22 (0.15)	
1985-1994	18110 (0.26)	14510 (0.27)	3600 (0.22)		97 (0.34)	43 (0.28)	
1995-2004	17455 (0.25)	12791 (0.24)	4664 (0.29)		64 (0.23)	54 (0.35)	
≥ 2005	20800 (0.29)	15073 (0.28)	5727 (0.35)		34 (0.12)	33 (0.22)	
**Race**				<0.001			0.003
White	59422 (0.85)	46263 (0.86)	13159 (0.81)		251 (0.89)	117 (0.77)	
Black	5093 (0.07)	3602 (0.07)	1491 (0.09)		12 (0.04)	17 (0.11)	
Other	5699 (0.08)	4118 (0.07)	1581 (0.10)		19 (0.07)	18 (0.12)	
**Tumor Stage**				<0.001			<0.001
Localized	46135 (0.66)	40561 (0.75)	5574 (0.34)		226 (0.80)	48 (0.32)	
Regional	24079 (0.34)	13422 (0.25)	10657(0.66)		56 (0.20)	104 (0.68)	
**Tumor Grade**				<0.001			0.638
Grade I/II	48707 (0.69)	38046 (0.70)	10661(0.66)		231 (0.82)	128 (0.84)	
Grade III/IV	21507 (0.31)	15937 (0.30)	5570 (0.34)		51 (0.18)	24 (0.16)	
**Tumor Size**				<0.001			0.009
<5	5141 (0.07)	4156 (0.08)	985 (0.06)		7 (0.03)	6 (0.04)	
≥5	16789 (0.24)	11684 (0.22)	5105 (0.31)		32 (0.11)	33 (0.22)	
Unknown	48284 (0.69)	38143 (0.70)	10141 (0.63)		243 (0.86)	113 (0.74)	
**Tumor Site**				<0.001			<0.001
Rectum and Rectosigmoid	27342 (0.39)	18740 (0.35)	8872 (0.55)		141 (0.50)	128 (0.84)	
Anus, Anal Canal and Anorectum	1624 (0.02)	548 (0.10)	1076 (0.07)		2 (0.01)	9 (0.06)	
Cervix Uteri	12031 (0.17)	7529 (0.14)	4502 (0.27)		3 (0.01)	10 (0.07)	
Ovary	10345 (0.15)	9990 (0.19)	355 (0.02)		24 (0.08)	1 (0.01)	
Bladder	18602 (0.27)	17176 (0.32)	1426 (0.09)		112 (0.40)	4 (0.02)	
**Chemotherapy**				<0.001			<0.001
No	50774 (0.72)	44776 (0.83)	5998 (0.37)		247 (0.88)	54 (0.35)	
Yes	19440 (0.28)	9207 (0.17)	10233 (0.63)		35 (0.12)	98 (0.65)	

P-value was calculated using the χ2 test for categorical variables.

PPC, primary pelvic cancers; NRT, no radiation therapy; RT, radiation therapy.

### Risk of RT for developing SCUC

**Figure 2:**
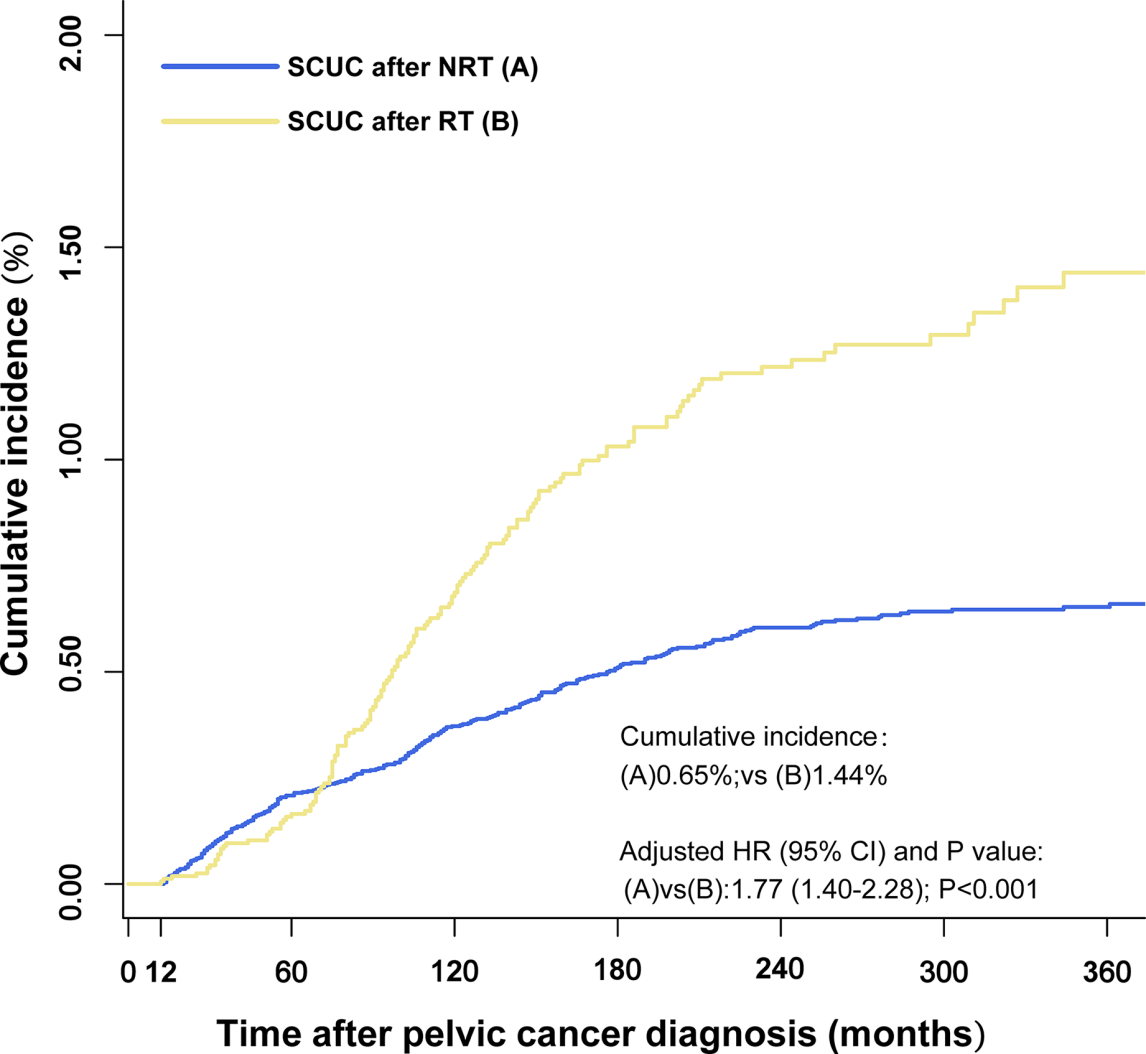
Comparisons of the cumulative incidence of secondary corpus uteri cancer (SCUC) between patients who received radiation therapy (RT) and patients who did not receive RT (NRT) in primary pelvic cancer (PPC) patients. The adjusted hazard ratios (HR) were adjusted for age at PPC diagnosis, tumor site, and chemotherapy by multivariable competing risk regression analysis. RT, radiation therapy; NRT, no radiation therapy; HR, hazard ratios.

**Figure 3:**
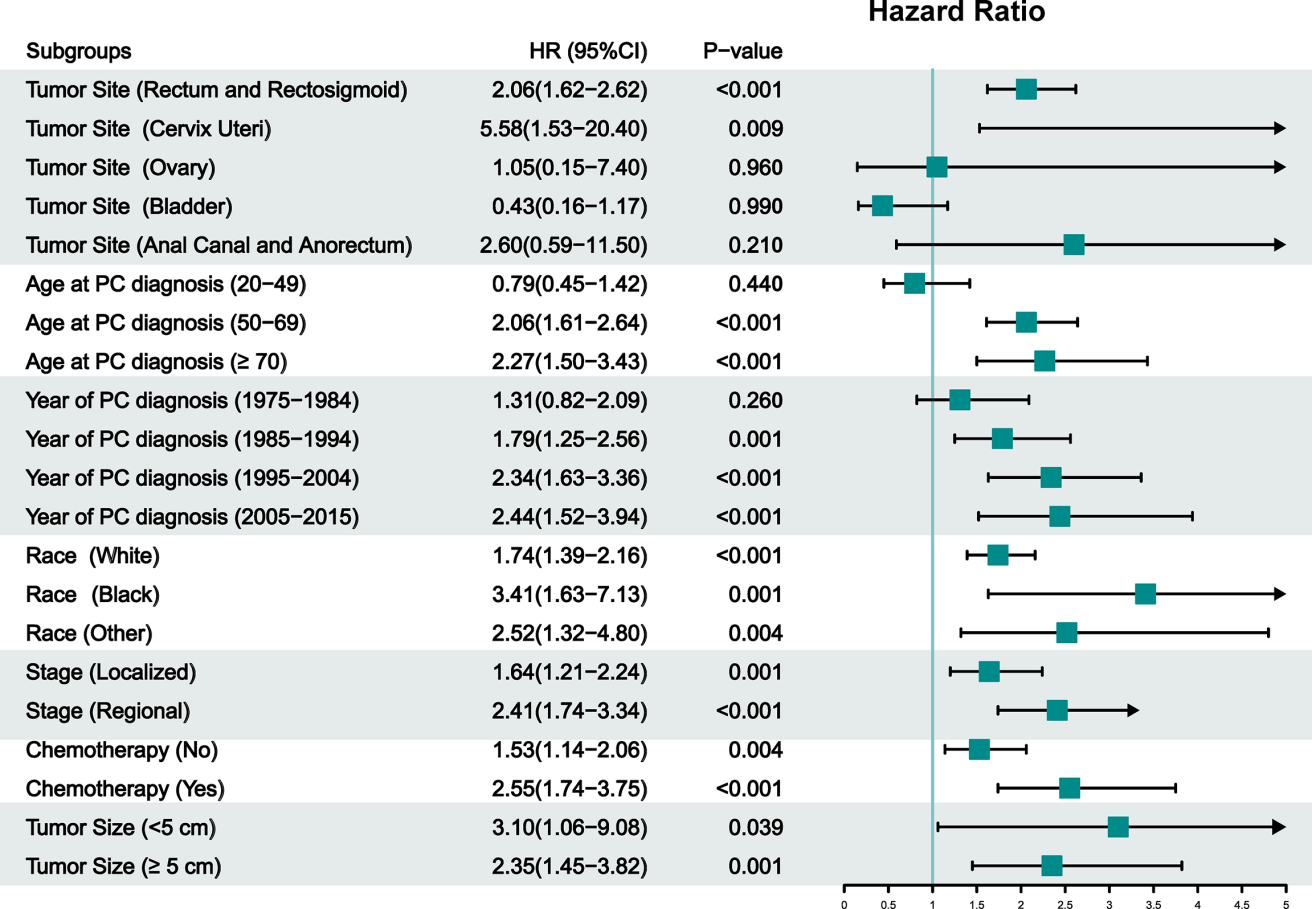
Subgroup analysis of competing risk regression analysis for the risk of developing secondary corpus uteri cancer (SCUC) in primary pelvic cancer (PPC) patients. RT, radiation therapy; NRT, no radiation therapy; PPC, primary pelvic cancers; CI, confidence interval; HR, hazard ratio.

The cumulative incidence of SCUC in the RT group (1.44%) is higher than that in the NRT group (0.65%) (adjusted HR = 1.77; 95% CI, 1.40–2.28; *p* < 0.001) (**Figure 2**). The important features were selected to evaluate the risk of developing SCUC in univariable competing risk regression (**Table 2**). It was demonstrated that RT for PPC and other variables including age at PPC diagnosis, and chemotherapy could significantly influence the risk of developing SCUC in univariable analysis, with *p* < 0.05. We then performed a multivariable analysis to further assess the role of these factors in the development of SCUC for PPC patients. Factors including RT, age at PPC diagnosis, tumor site, and chemotherapy could affect the risk of SCUC for PPC survivors. In the final multivariable analysis, RT for PPC was proved to be an independent risk factor for developing SCUC in PPC survivors (adjusted HR, 1.79; 95% CI, 1.40–2.28; adjusted *p* < 0.001). A Cox regression analysis was also performed to identify factors that could probably influence the occurrence of SCUC (**Supplementary Table S1**). Consistent with the results of competing risk regression, multivariable Cox regression analysis demonstrated that RT for PPC was an independent risk factor for SCUC (HR, 2.18; 95% CI, 1.67–2.87; *p* < 0.001). Moreover, subgroup analysis was also performed to further assess the risk of developing SCUC by competing for risk regression and found that RT for PPC was related to an increased risk of developing SCUC with statistical significance in most subgroups (**Figure 3**).

**Table 2:** Univariable and multivariable competing risk regression analyses of risk of developing SCUC in PPC patients.

Characteristic	Univariable analysis		Multivariable analysis	
	HR (95% CI)	*p*-value	HR (95% CI)	*p*-value
**Age at PPC diagnosis**				
20–49	Ref		Ref	
50–69	2.18 (1.66–2.85)	<0.001	1.19 (0.89–1.57)	0.230
≥70	0.99 (0.72–1.35)	0.960	0.50 (0.35–0.69)	<0.001
**Year of PPC diagnosis**				
1975–1984	Ref			
1985–1994	0.98 (0.77–1.27)	0.920		
1995–2004	0.91 (0.70–1.18)	0.470		
≥2005	0.80 (0.59–1.08)	0.150		
**Race**				
White	Ref			
Black	0.98 (0.67–1.43)	0.920		
Other	1.20 (0.85–1.68)	0.290		
**Tumor stage**				
Localized	Ref			
Regional	1.16 (0.95–1.41)	0.130		
**Tumor size**				
<5	Ref			
≥5	1.25 (0.68–2.27)	0.470		
Unknown	1.39 (0.79–2.42)	0.240		
**Tumor site**				
Rectum and rectosigmoid	Ref		Ref	
Anus, anal canal, and anorectum	0.74 (0.40–1.35)	0.330	0.60 (0.32–1.09)	0.093
Cervix uteri	0.12 (0.06–0.20)	<0.001	0.09 (0.05–0.17)	<0.001
Ovary	0.26 (0.17–0.39)	<0.001	0.28 (0.18–0.42)	<0.001
Bladder	0.58 (0.46–0.73)	<0.001	0.72 (0.57–0.89)	<0.001
**Chemotherapy**				
No	Ref		Ref	
Yes	1.38 (1.12–1.69)	<0.001	0.89 (0.68–1.14)	0.340
**Radiation**				
No	Ref		Ref	
Yes	1.89 (1.55–2.31)	<0.001	1.79 (1.40–2.28)	<0.001

Fine–gray competing risk regression analyses were used to calculate the hazard ratios (HRs) and 95% confidence intervals (CIs) for SCUC in pelvic cancer patients treated with RT versus patients not treated with RT. Covariables that are significant in univariable competing risk regression analysis (p < 0.050) are included in the multivariable analysis.

PPC, primary pelvic cancers; SCUC, second corpus uteri cancer; HR, hazard ratio; CI, confidence interval.

### Dynamic incidence risk for SCUC

**Figure 4:**
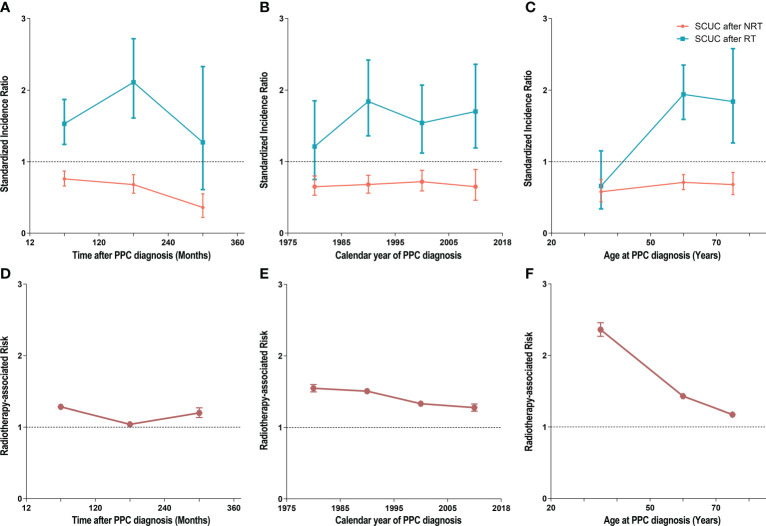
**(A)** Dynamic standardized incidence ratio (SIR) for secondary corpus uteri cancer (SCUC) in the latency-SIR plot. **(B)** Dynamic SIR for SCUC in PPC diagnosis time-SIR plot. **(C)** Dynamic SIR for SCUC in PPC diagnosis age-SIR plot. **(D)** Dynamic radiotherapy-associated risk (RR) for SCUC in the latency-RR plot. **(E)** Dynamic RR for SCUC in PPC diagnosis time-RR plot. **(F)** Dynamic RR for SCUC in PPC diagnosis age-RR plot. **(A–C)** Adjusted SIRs and 95% CIs of developing SCUC in primary pelvic cancer (PPC) patients treated with RT versus the US general population are plotted, as well as PPC patients who did not receive RT versus the US general population, and the incidence in the background US population is represented by the gray line (at *y* = 1). The detailed data of SIRs are shown in **Supplementary Table 2**. **(D–F)** The RR was estimated by Poisson regression analysis with the relative risks and 95% CIs of SCUC in primary PPC patients who underwent RT compared with those who did not undergo RT. The RR was adjusted for age at PPC diagnosis and calendar year of PPC diagnosis. In our study, the high risk of developing SCUC from RT required both adjusted HR >1 and adjusted RR >1, and the low risk of developing SLC from RT required both adjusted HR <1 and adjusted RR <1. RT, radiation therapy; NRT, no radiation therapy; RR, radiotherapy-associated risk; SIR, standardized incidence ratio; SCUC, secondary corpus uteri cancer; PPC, primary pelvic cancers.

We calculated the SIRs and RRs according to latency from PPC diagnosis, age at PPC diagnosis, and year of PPC diagnosis and established three dynamic plots to further evaluate the risk of SCUC for PPC patients treated with RT and without RT, respectively (**Figure 4**; **Supplementary Tables S2**, **S3**). In the dynamic latency-SIR plot, we found that the risk of developing SCUC increased with time after a 5-year latency from the diagnosis of PPC in the RT group but not in the NRT group (**Figure 4A**; **Supplementary Table S2**). In the dynamic latency-RR plot, although RR showed a trend of fluctuating downward on a whole (from 1.28 to 1.20), RR was greater than 1 with the increasing latent period (**Figure 4D**; **Supplementary Table S3**). Additionally, in the dynamic diagnosis time-SIR plot, we observed that the risk of SCUC increased with fluctuation for patients who received RT for their PPC as the calendar year of PPC diagnosis increased when compared with the background incidence rate of SCUC (**Figure 4B**). However, as for dynamic RR for the calendar year of PPC diagnosis, the gradual descent of RR was witnessed as the year of PPC diagnosis raised oppositely (**Figure 4E**; **Supplementary Table S3**). Furthermore, in the dynamic age-SIR plot, RT group patients presented a tendency for a decreasing risk of developing SCUC with the increasing age at PPC diagnosis, indicating that patients who were diagnosed with PPC at younger ages were at a higher risk of developing SCUC compared to older patients among PPC survivors who underwent RT (**Figure 4C**; **Supplementary Table S2**). The RR for age at PPC diagnosis was still more than 1, but the value of the RR decreased inversely with the increased in diagnostic age (**Figure 4F**; **Supplementary Table S3**).

### Survival outcome of SCUC

**Figure 5:**
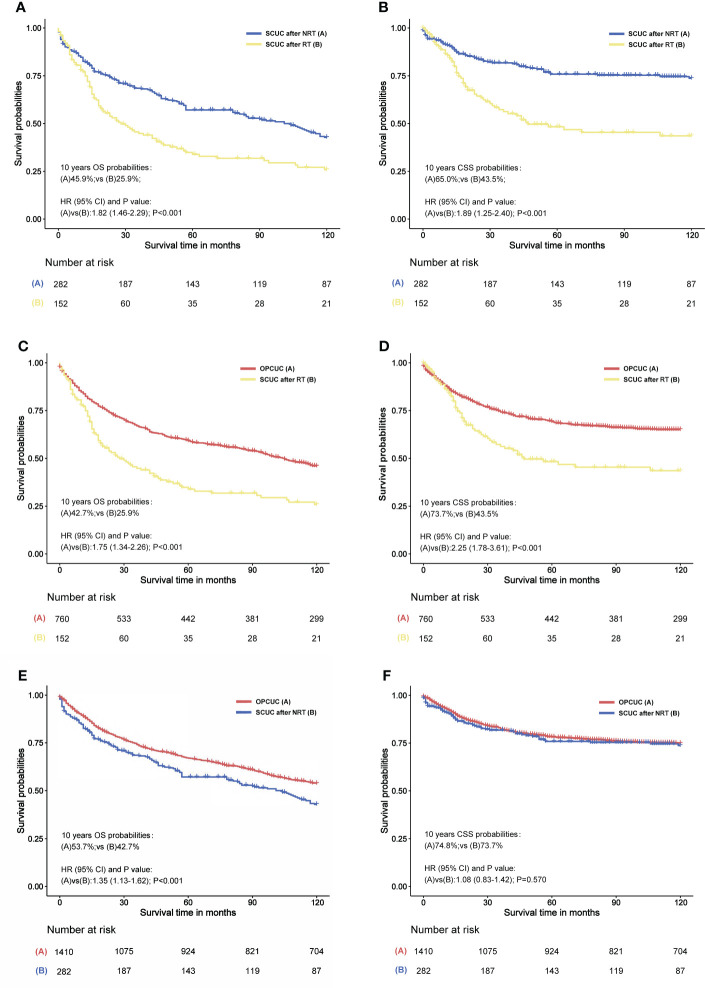
**(A)** Overall survival (OS) comparison between primary pelvic cancers (PPC) patients who developed secondary corpus uteri cancer (SCUC) after radiation therapy (RT) and PPC patients who developed SCUC after no RT (NRT). **(B)** Cancer-specific survival (CSS) comparison between PPC patients who developed SCUC after RT and PPC patients who developed PPC after NRT. **(C)** OS comparison between PPC patients who developed SCUC after RT and patients with only primary corpus uteri cancer (OPCUC). **(D)** CSS comparison between PPC patients who developed SCUC after RT and patients with OPCUC. **(E)** OS comparison between PPC patients who developed SCUC after NRT and patients with OPCUC. **(F)** CSS comparison between PPC patients who developed SCUC after NRT and patients with OPCUC. **(C–F)** Case-control comparisons, PPC patients who developed SCUC (case group) versus patients with OPCUC (control group), with a PSM ratio of 1:5 for SCUC versus OPCUC. The variables matched for PSM included age at CUC diagnosis, year of CUC diagnosis, race, stage of CUC, and type of treatment for CUC. The detailed patient characteristics of OPCUC before and after PSM are shown in **Supplementary Table S5**. HRs were calculated by Cox regression. HR, hazard ratio; OPCUC, only primary corpus uteri cancer; RT, radiation therapy; CI, confidence interval; SCUC, secondary corpus uteri cancer; OS, overall survival; CSS, cancer-specific survival.

Univariable and multivariable Cox regression analyses of prognostic factors for overall survival in patients with SCUC were conducted, indicating that radiation therapy for PPC was associated with worse overall survival in patients with SCUC, with statistical meanings (*p* < 0.001). The HRs of radiotherapy in the univariable and multivariable Cox regression analysis were 1.74 (95% CI, 1.34–2.26) and 1.59 (95% CI, 1.22–2.07), respectively (**Supplementary Table S5**). Moreover, survival analysis was performed to investigate the impact of RT on the prognosis of patients with SCUC in the RT and NRT groups. In terms of OS, the 10-year OS rate (45.9%) of the NRT group was higher than that (25.9%) of the RT group (**Figure 5A**). As for CSS, the 10-year CSS rate of patients treated with RT and without RT was 43.5% and 65.0%, respectively (**Figure 5B**). These results suggested that RT was associated with worse survival outcomes in terms of OS and CSS, indicating that radiation-associated SCUC had a poor prognosis. To further estimate the effects of RT on survival outcomes of SCUC, the only primary CUC (OPCUC) patients, who were referred to as the patients with sporadic corpus uteri cancer and without any second cancers during their follow-up time, were set as the control group. Compared with matched population controls with OPCUC, a significant difference of 10-year OS and CSS was observed between patients who developed SCUC after RT and matched OPCUC (10-year OS, 25.9% vs. 42.7%, *p* < 0.001, **Figure 5C**; 10-year CSS, 43.5% vs. 73.7%, *p* < 0.001, **Figure 5D**). Furthermore, we next compared survival outcomes of SCUC patients without RT with matched OPCUC. It was observed that OPCUC patients had a better prognosis than SCUC patients without RT in terms of 10-year OS (53.7% vs. 42.7%, *p* < 0.001, **Figure 5E**). The difference for 10-year CSS between SCUC patients in the RT group and OPCUC failed to reach a statistical significance (73.7% vs. 74.8%, *p* = 0.570, **Figure 5F**).

## Discussion

It is a well-known phenomenon that radiation exposure can lead to malignant neoplasms. Although SCUC incidence post-RT is only 0.5%–0.8%, according to previous reports (22), SCUC can significantly impair the survival outcomes of PPC survivors post-RT (23). To our best knowledge, our SEER-based study investigated the largest patient cohort and explored the relationship between pelvic RT for PPC and SCUC risk for the first time. Among the population-based cohort of 70,214 patients, our data confirm that patients who have previously undergone pelvic RT for PPC are at an increased risk of developing SCUC. Treatment with pelvic RT may contribute to an excess risk of SCUC. We also observed that patients treated with pelvic RT for PPC had higher SCUC incidence than the general population. The SIR of SCUC after pelvic RT appeared to increase with prolonged latency and decrease with diagnosis age. Lastly, our data indicate that SCUC after pelvic RT results in a worse prognosis than PPC patients without RT.

Consistent with our findings, previous reports have demonstrated that RT for PPC is associated with an increased risk of SCUC (23–25). However, these studies have several limitations. First, the sample size of patients was relatively small (24–26), which can bias the interpretation of results and impair statistical power. In addition, although SCUC can potentially develop after irradiation of tumors of several pelvic organs adjacent to the uterus, it is evident that most relevant studies attempted to estimate the risk of SCUC after irradiation of cervical cancer only (22–26). For the first time, our study comprehensively estimates the impact of RT on PPC, including both cervical cancer and noncervical cancers. Consistent with published studies, our findings suggest that RT for cervical cancer indeed elevates the risk of developing SCUC. Additionally, RT for noncervical cancers, including bladder, ovary, and colorectal cancer, is also associated with an increased incidence of SCUC.

We also innovatively analyzed the dynamic incidence of SCUC based on latency duration, age at SCUC diagnosis, and year of SCUC diagnosis. In comparison to the general population, the SCUC incidence increased with the extension of latency duration from the diagnosis of PPC in the RT group. However, this tendency was not observed in the NRT group, indicating that pelvic RT induced an excess risk of developing SCUC. These findings suggest that we need more prolonged surveillance for patients who receive RT for PPC. It is also of great interest that the SCUC risk decreased with the increasing calendar year of PPC diagnosis. The possible explanation can be the improvement of RT treatments for PPC, which increases radiation delivery accuracy to the tumor area and consistently minimizes exposure of uninvolved normal tissue. Moreover, we also observed that patients diagnosed with PPC at a younger age were at a higher risk of developing SCUC. Understandably, younger patients have a longer life expectancy after RT for PPC, which imposes a greater cancer risk for SCUC in this group.

Furthermore, we present the prognosis of pelvic RT-related SCUC in this study. We performed a survival analysis to compare the survival outcomes of SCUC after pelvic RT to those without RT. Depending on entities, RT was associated with worse survival outcomes in terms of OS and CSS, which was consistent with previous reports (23). Several factors may contribute to this poor prognosis. Studies have shown that patients who develop SCUC after RT are more likely to have aggressive histological subtypes than patients with sporadic SCUC (24–26). This indicates that pelvic RT might be regarded as a carcinogenic factor in the development of poorly differentiated SCUC. Furthermore, a large proportion of patients were diagnosed with advanced-stage disease after RT for PPC (23, 27, 28), probably resulting in a worse prognosis of SCUC. In comparison to the majority of sporadic SCUC patients whose clinical manifestations are vaginal bleeding and other clinical presentations which are relatively noticeable, the clinical manifestations of SCUC after RT for PPC can be atypical and imperceptible, such as the enlarged uterus, leading to delays in diagnosis. Radiation-induced cervical stenosis may prevent early symptoms of vaginal bleeding. Thus, patients with SCUC might merely present with nonspecific symptoms of an enlarged uterus and abdominal pain or cramping, leading to a delay in diagnosis. Consequently, consideration should be given to long-term surveillance for SCUC in patients treated with RT for PPC. It is also crucial to educate patients to report any relevant symptoms to their clinicians, thereby facilitating early diagnosis of SCUC. Moreover, developments in treatments for early carcinoma in corpus uteri will also improve the prognosis and quality of patients’ lives (29, 30).

The greatest strengths of the present study are the large patient numbers and long-term follow-up of over 30 years, which provides higher statistical power and makes our study more generalizable. Moreover, instead of using COX regression, which is usually used to assess the risk of SCUC after pelvic RT, we used Fine–Gray competing risk regression analysis, thus avoiding interferences caused by competing events, including all-course death.

However, we must acknowledge certain limitations of this study. Foremost, this was a retrospective study that prevented treatment randomization and increased the potential for confounding. Additionally, other risk factors that may alter the risk of SCUC, including genetic predisposition, lifestyle, family history, and environmental factors, were not available in the SEER database. Confounding can happen if these unmeasured covariates related to PPC were also associated with the development of SCUC. Hence, we used a multivariable competing model to adjust risk factors that were available, such as age at diagnosis and year of diagnosis. This study also lacks data on dosage, fractionation, and duration of RT because such information was not coded in the SEER database. Considering the significant advances in RT for PPC, RT modality has changed towards hypofractionation, which may influence the incidence of SCUC after RT. Therefore, more studies with randomized allocation and precise information on RT regimens are warranted. In addition, the precise proportion of patients had corpus uteri left after treatment for primary pelvic cancer was unknown, because the SEER database failed to provide detailed information (including surgical procedure and resection range) of surgical treatment. The treatment information we could obtain was whether patients received surgery, radiotherapy, or chemotherapy. Therefore, it was regarded as one of the limitations of this study.

In conclusion, we observed a higher risk of SCUC in patients who underwent RT for PPC compared to PPC patients without RT and the general US population. Moreover, the radiation-associated SCUC carried a grave prognosis. Therefore, long-lasting surveillance for patients receiving RT for PPC is recommended, especially for younger patients.

## Data availability statement

The original contributions presented in the study are included in the article/**Supplementary Material**. Further inquiries can be directed to the corresponding authors.

## Ethics statement

Written informed consent was obtained from the individual(s) for the publication of any potentially identifiable images or data included in this article.

## Author contributions

Manuscript writing: GY, RW, ZJ, XG. All authors contributed to the article and approved the submitted version.

## Conflict of interest

The authors declare that the research was conducted in the absence of any commercial or financial relationships that could be construed as a potential conflict of interest.

## Publisher’s note

All claims expressed in this article are solely those of the authors and do not necessarily represent those of their affiliated organizations, or those of the publisher, the editors and the reviewers. Any product that may be evaluated in this article, or claim that may be made by its manufacturer, is not guaranteed or endorsed by the publisher.

## References

[1] Delaney G Jacob S Featherstone C Barton M. The role of radiotherapy in cancer treatment: Estimating optimal utilization from a review of evidence-based clinical guidelines. Cancer (2005) 104:1129–37. doi: 10.1002/cncr.21324 16080176

[2] Begg AC Stewart FA Vens C. Strategies to improve radiotherapy with targeted drugs. Nat Rev Cancer (2011) 11:239–53. doi: 10.1038/nrc3007 21430696

[3] Baskar R Dai J Wenlong N Yeo R Yeoh K-W. Biological response of cancer cells to radiation treatment. Front Mol Biosci (2014) 1:24. doi: 10.3389/fmolb.2014.00024 25988165 PMC4429645

[4] Michalski JM Yan Y Watkins-Bruner D Bosch WR Winter K Galvin JM. Preliminary toxicity analysis of 3-dimensional conformal radiation therapy versus intensity modulated radiation therapy on the high-dose arm of the radiation therapy oncology group 0126 prostate cancer trial. Int J Radiat Oncol Biol Phys (2013) 87:932–8. doi: 10.1016/j.ijrobp.2013.07.041 PMC3840044 24113055

[5] Yeboa DN Evans SB. Contemporary breast radiotherapy and cardiac toxicity. Semin Radiat Oncol (2016) 26:71–8. doi: 10.1016/j.semradonc.2015.09.003 26617212

[6] Marijnen CAM Kapiteijn E van de Velde CJH Martijn H Steup WH Wiggers T. Acute side effects and complications after short-term preoperative radiotherapy combined with total mesorectal excision in primary rectal cancer: Report of a multicenter randomized trial. J Clin Oncol (2002) 20:817–25. doi: 10.1200/JCO.2002.20.3.817 11821466

[7] Maduro JH Pras E Willemse PHB de Vries EGE. Acute and long-term toxicity following radiotherapy alone or in combination with chemotherapy for locally advanced cervical cancer. Cancer Treat Rev (2003) 29:471–88. doi: 10.1016/S0305-7372(03)00117-8 14585258

[8] Schmid MP Pötter R Bombosch V Sljivic S Kirisits C Dörr W. Late gastrointestinal and urogenital side-effects after radiotherapy–incidence and prevalence. subgroup-analysis within the prospective Austrian-German phase II multicenter trial for localized prostate cancer. Radiother Oncol (2012) 104:114–8. doi: 10.1016/j.radonc.2012.05.007 22727264

[9] Rombouts AJM Hugen N van Beek JJP Poortmans PMP de Wilt JHW Nagtegaal ID. Does pelvic radiation increase rectal cancer incidence? - a systematic review and meta-analysis. Cancer Treat Rev (2018) 68:136–44. doi: 10.1016/j.ctrv.2018.05.008 29957373

[10] Warschkow R Güller U Cerny T Schmied BM Plasswilm L Putora PM. Secondary malignancies after rectal cancer resection with and without radiation therapy: A propensity-adjusted, population-based SEER analysis. Radiother Oncol (2017) 123:139–46. doi: 10.1016/j.radonc.2017.02.007 28285840

[11] Ohno T Kato S Sato S Fukuhisa K Nakano T Tsujii H. Long-term survival and risk of second cancers after radiotherapy for cervical cancer. Int J Radiat Oncol Biol Phys (2007) 69:740–5. doi: 10.1016/j.ijrobp.2007.04.028 17889265

[12] Liauw SL Sylvester JE Morris CG Blasko JC Grimm PD. Second malignancies after prostate brachytherapy: Incidence of bladder and colorectal cancers in patients with 15 years of potential follow-up. Int J Radiat Oncol Biol Phys (2006) 66:669–73. doi: 10.1016/j.ijrobp.2006.05.016 16887293

[13] Hird AE Magee DE Matta R Saskin R Dvorani E Kulkarni GS. Assessment of secondary sarcomas among patients with cancer of the abdomen or pelvis who received combinations of surgery, radiation, and chemotherapy vs surgery alone. JAMA Netw Open (2020) 3:e2013929. doi: 10.1001/jamanetworkopen.2020.13929 33006617 PMC7532387

[14] Okajima K Ishikawa K Matsuura T Tatebe H Fujiwara K Hiroi K. Multiple primary malignancies in patients with prostate cancer: Increased risk of secondary malignancies after radiotherapy. Int J Clin Oncol (2013) 18:1078–84. doi: 10.1007/s10147-012-0496-3 23179638

[15] Vuolukka K Auvinen P Palmgren J-E Aaltomaa S Kataja V. Incidence of subsequent primary cancers and radiation-induced subsequent primary cancers after low dose-rate brachytherapy monotherapy for prostate cancer in long-term follow-up. BMC Cancer (2020) 20:453. doi: 10.1186/s12885-020-06960-9 32434560 PMC7240976

[16] Birgisson H Påhlman L Gunnarsson U Glimelius B. Occurrence of second cancers in patients treated with radiotherapy for rectal cancer. J Clin Oncol (2005) 23:6126–31. doi: 10.1200/JCO.2005.02.543 16135478

[17] Rombouts AJM Hugen N Elferink MAG Feuth T Poortmans PMP Nagtegaal ID. Incidence of second tumors after treatment with or without radiation for rectal cancer. Ann Oncol (2017) 28:535–40. doi: 10.1093/annonc/mdw661 27993790

[18] Abdel-Wahab M Reis IM Hamilton K. Second primary cancer after radiotherapy for prostate cancer–a seer analysis of brachytherapy versus external beam radiotherapy. Int J Radiat Oncol Biol Phys (2008) 72:58–68. doi: 10.1016/j.ijrobp.2007.12.043 18374503

[19] Rombouts AJM Hugen N Elferink MAG Poortmans PMP Nagtegaal ID de Wilt JHW. Increased risk for second primary rectal cancer after pelvic radiation therapy. Eur J Cancer (2020) 124:142–51. doi: 10.1016/j.ejca.2019.10.022 31765989

[20] Hinnen KA Schaapveld M van Vulpen M Battermann JJ van der Poel H van Oort IM. Prostate brachytherapy and second primary cancer risk: A competitive risk analysis. J Clin Oncol (2011) 29:4510–5. doi: 10.1200/JCO.2011.35.0991 22025166

[21] Kumar S Shah JP Bryant CS Seward S Ali-Fehmi R Morris RT. Radiation-associated endometrial cancer. Obstet Gynecol (2009) 113:319–25. doi: 10.1097/AOG.0b013e3181954c5b 19155901

[22] Gallion HH van Nagell JR Donaldson ES Powell DE. Endometrial cancer following radiation therapy for cervical cancer. Gynecol Oncol (1987) 27:76–83. doi: 10.1016/0090-8258(87)90232-0 3570051

[23] Pothuri B Ramondetta L Martino M Alektiar K Eifel PJ Deavers MT. Development of endometrial cancer after radiation treatment for cervical carcinoma. Obstet Gynecol (2003) 101:941–5. doi: 10.1016/s0029-7844(03)00234-5 12738155

[24] Behtash N Tehranian A Ardalan FA Hanjani P. Uterine papillary serous carcinoma after pelvic radiation therapy for cancer of the cervix. J Obstet Gynaecol (2002) 22:96–7. doi: 10.1080/01443610211114 12521747

[25] Park MH Cho SH Kang HJ Kim SR Hwang YY. Uterine papillary serous carcinoma following radiation therapy for carcinoma of cervix: A case report. Int J Gynecol Cancer (2000) 10:253–6. doi: 10.1046/j.1525-1438.2000.010003253.x 11240683

[26] Pothuri B Ramondetta L Eifel P Deavers MT Wilton A Alektiar K. Radiation-associated endometrial cancers are prognostically unfavorable tumors: A clinicopathologic comparison with 527 sporadic endometrial cancers. Gynecol Oncol (2006) 103:948–51. doi: 10.1016/j.ygyno.2006.05.039 16870239

[27] Rodriguez J Hart WR. Endometrial cancers occurring 10 or more years after pelvic irradiation for carcinoma. Int J Gynecol Pathol (1982) 1:135–44. doi: 10.1097/00004347-198202000-00002 7184892

[28] Fehr PE Prem KA. Malignancy of the uterine corpus following irradiation therapy for squamous cell carcinoma of the cervix. Am J Obstet Gynecol (1974) 119:685–92. doi: 10.1016/0002-9378(74)90133-1 4365384

[29] Giampaolino P Di Spiezio Sardo A Mollo A Raffone A Travaglino A Boccellino A. Hysteroscopic endometrial focal resection followed by levonorgestrel intrauterine device insertion as a fertility-sparing treatment of atypical endometrial hyperplasia and early endometrial cancer: A retrospective study. J Minim Invasive Gynecol (2019) 26:648–56. doi: 10.1016/j.jmig.2018.07.001 30017893

[30] L. Della Corte Giampaolino P Mercorio A Riemma G Schiattarella A De Franciscis P. Sentinel lymph node biopsy in endometrial cancer: State of the art. Transl Cancer Res (2020) 9:7725–33. doi: 10.21037/tcr.2020.04.21 PMC8797296 35117375

