# Supplementary material for: Risk and prognosis of second corpus uteri cancer after radiation therapy for pelvic cancer: A population-based analysis
Source: Front Oncol. 2022 Sep 29;12:957608. doi: 10.3389/fonc.2022.957608 (PMC9556627; doi:10.3389/fonc.2022.957608)
Supplement: Supplementary file 1 [file Table_1.docx]

**(Supplementary)TABLE 1.** Univariable and Multivariable Cox Regression Analysis of Risk of Developing SCUC in PPC Patients.

| **Characteristic** | **Univariable Analysis** | | **Multivariable Analysis** | |
| --- | --- | --- | --- | --- |
|  | HR (95%Cl) | P value | HR (95%Cl) | P value |
| **Tumor Site** |  |  |  |  |
| Rectum and Rectosigmoid | Ref |  |  |  |
| Anus, Anal Canal and Anorectum | 0.73 (0.41-1.34) | 0.318 | 0.57 (0.31-1.05) | 0.073 |
| Cervix Uteri | 0.08 (0.04-0.13) | <0.001 | 0.08 (0.04-0.15) | <0.001 |
| Ovary | 0.19 (0.13-0.29) | <0.001 | 0.26 (0.17-0.41) | <0.001 |
| Bladder | 0.56 (0.45-0.70) | <0.001 | 0.72 (0.57-0.91) | 0.007 |
| **Age at PPC Diagnosis** |  |  |  |  |
| 20-49 | Ref |  | Ref |  |
| 50-69 | 2.9 (2.21-3.80) | <0.001 | 1.51 (1.14-2.01) | 0.003 |
| ≥ 70 | 2.29 (1.67-3.14) | <0.001 | 1.12 (0.81-1.55) | 0.492 |
| **Year of PPC Diagnosis** |  |  |  |  |
| 1975-1984 | Ref |  |  |  |
| 1985-1994 | 0.99 (0.77-1.28) | 0.950 |  |  |
| 1995-2004 | 0.93 (0.71-1.21) | 0.593 |  |  |
| ≥ 2005 | 1.02 (0.74-1.40) | 0.905 |  |  |
| **Race** |  |  |  |  |
| White | Ref |  |  |  |
| Black | 1.05 (0.71-1.53) | 0.803 |  |  |
| Other | 1.09 (0.78-1.53) | 0.602 |  |  |
| **Tumor Stage** |  |  |  |  |
| Localized | Ref |  | Ref |  |
| Regional | 1.71 (1.41-2.072) | <0.001 | 1.05 (0.84-1.32) | 0.625 |
| **Chemotherapy** |  |  |  |  |
| No | Ref |  | Ref |  |
| Yes | 1.54 (1.25-1.891) | <0.001 | 0.93 (0.71-1.22) | 0.596 |
| **Radiation** |  |  |  |  |
| No | Ref |  | Ref |  |
| Yes | 2.23 (1.83-2.719) | <0.001 | 2.18 (1.67-2.87) | <0.001 |
| **Tumor Size** |  |  |  |  |
| <5 | Ref |  |  |  |
| ≥ 5 | 1.51 (0.82-2.728) | 0.179 |  |  |
| Unknown | 1.29 (0.74-2.26) | 0.369 |  |  |

**NOTE:** Cox regression analysis were used to calculate the hazard ratios (HRs) and 95% confidence intervals (CIs) for SCUC in pelvic cancers patients treated with RT versus patients without RT. Covariables that are significant in univariable competing risk regression analysis (P<0.050) are included in the multivariable analysis.

**Abbreviations:** PPC, primary pelvic cancers; SCUC, second corpus uteri cancer; HR, hazard ratio; CI, confidence interval.

**(Supplementary)TABLE 2.** Standardized Incidence Ratio of SCUC by Age at PPC Diagnosis, Latency, Year of PPC Diagnosis, Race and Tumor Site of PPC.

| **Characteristic** | **RT vs US General Population** | **NRT vs US General Population** |
| --- | --- | --- |
|  | Adjusted SIR (95% CI) | Adjusted SIR (95% CI) |
| **All** | 1.66^#^ (1.41-1.93) | 0.68^#^ (0.61-0.75) |
| **Age at PPC Diagnosis** |  |  |
| 20-49 | 0.66 (0.34-1.15) | 0.58^#^ (0.44-0.75) |
| 50-69 | 1.94^#^ (1.59-2.35) | 0.71^#^ (0.61-0.82) |
| ≥ 70 | 1.84^#^ (1.26-2.58) | 0.68^#^ (0.54-0.85) |
| **Year of PPC Diagnosis** |  |  |
| 1975-1984 | 1.21 (0.75-1.85) | 0.65^#^ (0.53-0.80) |
| 1985-1994 | 1.84^#^ (1.36-2.42) | 0.68^#^ (0.56-0.81) |
| 1995-2004 | 1.54^#^ (1.12-2.07) | 0.72^#^ (0.59-0.88) |
| ≥ 2005 | 1.70^#^ (1.19-2.36) | 0.65^#^ (0.46-0.89) |
| **Race** |  |  |
| White | 1.51^#^ (1.26-1.80) | 0.68^#^ (0.61-0.76) |
| Black | 2.40^#^ (1.37-3.90) | 0.53^#^ (0.28-0.91) |
| Other | 2.88^#^ (1.71-4.55) | 0.79 (0.47-1.23) |
| **Tumor Site** |  |  |
| Rectum and Rectosigmoid | 2.33^#^ (1.97-2.73) | 1.01 (0.86-1.18) |
| Anus, Anal Canal and Anorectum | 1.35 (0.68-2.42) | 0.72 (0.15-2.10) |
| Cervix Uteri | 0.36^#^ (0.19-0.63) | 0.05^#^ (0.01-0.14) |
| Ovary | 0.22 (0.01-1.23) | 0.28^#^ (0.18-0.41) |
| Bladder | 0.71 (0.23-1.65) | 0.80^#^ (0.68-0.93) |
| **Latency** |  |  |
| 12-119 Months | 1.53^#^ (1.24-1.87) | 0.76^#^ (0.66-0.87) |
| 120-239 Months | 2.11^#^ (1.61-2.72) | 0.68^#^ (0.56-0.82) |
| 240-360 Months | 1.27 (0.61-2.33) | 0.36^#^ (0.22-0.55) |

**NOTE:** SIR was defined as the ratio of the number of observed secondary corpus uteri cancer (SCUC) cases among primary pelvic cancers (PPC) survivors to the expected number of cases in the US general population and was stratified by age at PPC diagnosis and calendar year of PPC diagnosis. A determination of the statistical significance of SIRs was based on a P<0.05 (two sided). 95% confidence intervals were calculated by Poisson exact methods. The background incidence of SCUC was derived from data provided by the SEER database.

**Abbreviations:** PPC, primary pelvic cancers; SCUC, second corpus uteri cancer; SIR, standardized incidence ratios; CI, confidence interval; NRT, no radiation therapy; RT, radiation therapy

^#^ p < 0.05.

**(Supplementary)TABLE 3.** Radiation-attributed Risk of Secondary Corpus Uteri Cancer by Age at PPC Diagnosis, Latency, Year of PPC Diagnosis, Race and Tumor Site of PPC.

| **Characteristic** | **Univariable Analysis** | | **Multivariable Analysis** | |
| --- | --- | --- | --- | --- |
|  | RR (95%Cl) | P-value | RR (95%Cl) | P-value |
| **All** | 1.21 (1.19-1.23) | <0.001 | 1.42 (1.40-1.44) | <0.001 |
| **Age at PPC Diagnosis** |  |  |  |  |
| 20-49 | 2.29 (2.20-2.38) | <0.001 | 2.36 (2.27-2.46) | <0.001 |
| 50-69 | 1.32 (1.29-1.36) | <0.001 | 1.43 (1.40-1.46) | <0.001 |
| ≥ 70 | 1.14 (1.11-1.17) | <0.001 | 1.17 (1.14-1.20) | <0.001 |
| **Year of PPC Diagnosis** |  |  |  |  |
| 1975-1984 | 1.37 (1.32-1.41) | <0.001 | 1.55 (1.50-1.60) | <0.001 |
| 1985-1994 | 1.33 (1.30-1.37) | <0.001 | 1.51(1.46-1.55) | <0.001 |
| 1995-2004 | 1.15 (1.12-1.19) | <0.001 | 1.33 (1.29-1.37) | <0.001 |
| ≥ 2005 | 1.18 (1.13-1.22) | <0.001 | 1.28 (1.23-1.33) | <0.001 |
| **Race** |  |  |  |  |
| White | 1.19 (1.17-1.21) | <0.001 | 1.40 (1.38-1.43) | <0.001 |
| Black | 1.22 (1.16-1.30) | <0.001 | 1.38 (1.31-1.46) | <0.001 |
| Other | 1.50 (1.41-1.59) | <0.001 | 1.61 (1.51-1.70) | <0.001 |
| **Tumor Site** |  |  |  |  |
| Rectum and Rectosigmoid | 1.01 (0.98-1.03) | 0.575 | 1.30 (1.27-1.33) | <0.001 |
| Anus, Anal Canal and Anorectum | 0.77 (0.70-0.84) | <0.001 | 0.94 (0.86-1.04) | 0.239 |
| Cervix Uteri | 2.59 (2.48-2.70) | <0.001 | 2.17 (2.08-2.27) | <0.001 |
| Ovary | 1.66 (1.52-1.80) | <0.001 | 1.60 (1.47-1.74) | <0.001 |
| Bladder | 2.11 (2.03-2.20) | <0.001 | 1.87 (1.80-1.95) | <0.001 |
| **Latency** |  |  |  |  |
| 12-119 Months | 1.08 (1.06-1.10) | <0.001 | 1.28 (1.26-1.31) | <0.001 |
| 120-239 Months | 0.80 (0.77-0.82) | <0.001 | 1.04 (1.00-1.08) | 0.036 |
| 240-360 Months | 0.97 (0.92-1.03) | 0.338 | 1.20 (1.14-1.27) | <0.001 |

**NOTE:** Poisson regression analysis were used to calculate the radiation-attributed risk (RR) and 95% confidence intervals (CIs) of SCUC for patients with radiation therapy versus patients without radiation therapy. Adjusted RRs were stratified by age at primary pelvic cancers (PPC) diagnosis and calendar year of PPC diagnosis. A determination of statistical significance of RRs was based on P<0.05 (two sided).

**Abbreviations:** PPC, primary pelvic cancers; SCUC, second corpus uteri cancer; SIR, standardized incidence ratios; CI, confidence interval; NRT, no radiation therapy; RT, radiation therapy

**(Supplementary)TABLE 4.** Comparisons of Baseline Characteristics of Patients with SCUC by Treatment Modality

| **Characteristic** | **NRT (N=282)** | **RT (N=152)** | **P-value** |
| --- | --- | --- | --- |
| **Age at PPC Diagnosis** |  |  | 0.006 |
| 20-49 | 26 (0.10) | 3 (0.02) |  |
| 50-69 | 95 (0.33) | 66 (0.43) |  |
| ≥ 70 | 161 (0.57) | 83 (0.55) |  |
| **Year of PPC Diagnosis** |  |  | <0.001 |
| 1975-1984 | 22 (0.08) | 3 (0.02) |  |
| 1985-1994 | 69 (0.24) | 20 (0.13) |  |
| 1995-2004 | 88 (0.31) | 40 (0.26) |  |
| ≥ 2005 | 103 (0.37) | 89 (0.59) |  |
| **Race** |  |  | 0.003 |
| White | 251 (0.89) | 117 (0.77) |  |
| Black | 12 (0.04) | 17 (0.11) |  |
| Other | 19 (0.07) | 18 (0.12) |  |
| **Tumor Stage** |  |  | <0.001 |
| Localized | 177 (0.63) | 58 (0.38) |  |
| Regional | 46 (0.16) | 40 (0.26) |  |
| Distant | 25 (0.09) | 19 (0.13) |  |
| Unknown | 34 (0.12) | 35 (0.23) |  |
| **Tumor Grade** |  |  | <0.001 |
| Grade I/II | 144 (0.51) | 31 (0.20) |  |
| Grade III/IV | 81 (0.29) | 71 (0.47) |  |
| Unknown | 57 (0.20) | 50 (0.33) |  |
| **Chemotherapy** |  |  | <0.001 |
| No | 244 (0.87) | 102 (0.67) |  |
| Yes | 38 (0.13) | 50 (0.33) |  |
| **Surgery** |  |  | 1.00 |
| No | 40 (0.14) | 21 (0.14) |  |
| Yes | 242 (0.86) | 131 (0.86) |  |
| **Radiation Therapy** |  |  | 0.003 |
| No | 196 (0.70) | 126 (0.83) |  |
| Yes | 86 (0.30) | 26 (0.17) |  |

**NOTE:** P-value was calculated using the χ^2^ test (^b^) for categorical variables.

**Abbreviations:** PPC, primary pelvic cancers; SCUC, second corpus uteri cancer; NRT, no radiation therapy; RT, radiation therapy.

**(Supplementary)TABLE 5.** Univariable and Multivariable Cox Regression Analysis of Prognostic Factors for Overall Survival in Patients with SCUC.

| **Characteristic** | **Univariable Analysis** | | **Multivariable Analysis** | |
| --- | --- | --- | --- | --- |
|  | HR (95%Cl) | P-value | HR (95%Cl) | P-value |
| **Age at SCUC Diagnosis** |  |  |  |  |
| 20-49 | Ref |  | Ref |  |
| 50-69 | 2.42 (1.05-5.57) | 0.038 | 2.37 (1.02-5.49) | 0.044 |
| ≥ 70 | 4.95 (2.19-11.19) | <0.001 | 4.46 (1.97-10.10) | <0.001 |
| **Year of SCUC Diagnosis** |  |  |  |  |
| 1975-1984 | Ref |  |  |  |
| 1985-1994 | 1.34 (0.72-2.51) | 0.355 |  |  |
| 1995-2004 | 1.40 (0.76-2.57) | 0.278 |  |  |
| ≥ 2005 | 1.49 (0.81-2.72) | 0.199 |  |  |
| **Race** |  |  |  |  |
| White | Ref |  |  |  |
| Black | 1.00 (0.57-1.75) | 0.999 |  |  |
| Other | 1.23 (0.79-1.93) | 0.362 |  |  |
| **Tumor Stage** |  |  |  |  |
| Localized | Ref |  |  |  |
| Regional | 1.34 (0.72-2.51) | 0.355 |  |  |
| Distant | 1.40 (0.76-2.57) | 0.278 |  |  |
| Unknown | 1.49 (0.81-2.72) | 0.199 |  |  |
| **Chemotherapy for SCUC** |  |  |  |  |
| No | Ref |  |  |  |
| Yes | 0.98 (0.70-1.38) | 0.903 |  |  |
| **Surgery for SCUC** |  |  |  |  |
| No | Ref |  | Ref |  |
| Yes | 0.27 (0.20-0.38) | <0.001 | 0.33 (0.23-0.45) | <0.001 |
| **Radiation Therapy for PPC** |  |  |  |  |
| No | Ref |  | Ref |  |
| Yes | 1.74 (1.34-2.26) | <0.001 | 1.59 (1.22-2.07) | <0.001 |
| **Radiation Therapy for SCUC** |  |  |  |  |
| No | Ref |  |  |  |
| Yes | 0.77 (0.57-1.03) | 0.081 |  |  |

**NOTE:** Cox regression analysis were used to calculate the hazard ratios (HRs) and 95% confidence intervals (CIs) for overall survival in patients with SCUC. Covariables that are significant in univariable cox regression analysis (P<0.050) are included in the multivariable analysis.

**Abbreviations:** PPC, primary pelvic cancers; SCUC, second corpus uteri cancer; HR, hazard ratio; CI, confidence interval.

**Supplementary Table 6.** Comparisons of Baseline Characteristics of Patients with SCUC and Patients with OPCUC.

| **Characteristic** | **SCUC (NRT)** | **OPCUC Matched With SCUC (NRT)** | **P-value** | **SCUC (RT)** | **OPCUC Matched With SCUC (RT)** | **P-value** |  |
| --- | --- | --- | --- | --- | --- | --- | --- |
|  | n=760 | n=152 |  | n=1410 | n=282 |  |  |
|  |  |  |  |  |  |  |  |
| **Age at CUC Diagnosis** |  |  | 1.000 |  |  | 1.000 |  |
| 20-49 | 15 (2.0) | 3 (2.0) |  | 130 (9.2) | 26 (9.2) |  |  |
| 50-69 | 331 (43.5) | 66 (43.4) |  | 475 (33.7) | 95 (33.7) |  |  |
| ≥ 70 | 414 (54.5) | 83 (54.6) |  | 805 (57.1) | 161 (57.1) |  |  |
| **Year of CUC Diagnosis** |  |  | 0.999 |  |  | 0.998 |  |
| 1975-1984 | 15 (2.0) | 3 (2.0) |  | 110 (7.8) | 22 (7.8) |  |  |
| 1985-1994 | 103 (13.6) | 20 (13.2) |  | 338 (24.0) | 69 (24.5) |  |  |
| 1995-2004 | 196 (25.8) | 40 (26.3) |  | 442 (31.3) | 88 (31.2) |  |  |
| ≥ 2005 | 446 (58.7) | 89 (58.6) |  | 520 (36.9) | 103 (36.5) |  |  |
| **Race** |  |  | 0.999 |  |  | 0.987 |  |
| White | 584 (76.8) | 117 (77.0) |  | 1253 (88.9) | 251 (89.0) |  |  |
| Black | 85 (11.2) | 17 (11.2) |  | 63 (4.5) | 12 (4.3) |  |  |
| Other | 91 (12.0) | 18 (11.8) |  | 94 (6.7) | 19 (6.7) |  |  |
| **Tumor Stage** |  |  | 0.998 |  |  | 0.997 |  |
| Localized | 346 (45.5) | 70 (46.1) |  | 940 (66.7) | 188 (66.7) |  |  |
| Regional | 265 (34.9) | 53 (34.9) |  | 259 (18.4) | 51 (18.1) |  |  |
| Distant | 106 (13.9) | 21 (13.8) |  | 120 (8.5) | 25 (8.9) |  |  |
| Unknown | 43 (5.7) | 8 (5.3) |  | 91 (6.5) | 18 (6.4) |  |  |
| **Chemotherapy** |  |  | 1.000 |  |  | 1.000 |  |
| No | 510 (67.1) | 102 (67.1) |  | 1218 (86.4) | 244 (86.5) |  |  |
| Yes | 250 (32.9) | 50 (32.9) |  | 192 (13.6) | 38 (13.5) |  |  |
| **Surgery** |  |  | 1.000 |  |  | 0.900 |  |
| No | 102 (13.4) | 21 (13.8) |  | 193 (13.7) | 40 (14.2) |  |  |
| Yes | 658 (86.6) | 131 (86.2) |  | 1217 (86.3) | 242 (85.8) |  |  |
| **Radiation Therapy** |  |  | 1.000 |  |  | 1.000 |  |
| No | 630 (82.9) | 126 (82.9) |  | 981 (69.6) | 196 (69.5) |  |  |
| Yes | 130 (17.1) | 26 (17.1) |  | 429 (30.4) | 86 (30.5) |  |  |

**NOTE:** Primary pelvic cancers patients who developed second corpus uteri cancer (SCUC) were matched to patients with only primary corpus uteri cancer (OPCUC), with a PSM ratio of 1:5 for SCUC versus OPCUC patients. The variables matched for PSM included age at CUC diagnosis, year of CUC diagnosis, race, stage of CUC and type of treatments for corpus uteri cancer.

**Abbreviations:** PPC, primary pelvic cancers; NRT, no radiation therapy; RT, radiation therapy; SCUC, second corpus uteri cancer; OPCUC, only primary corpus uteri cancer; CUC, corpus uteri cancer.
